# Collagen abundance controls melanoma phenotypes through lineage-specific microenvironment sensing

**DOI:** 10.1038/s41388-018-0209-0

**Published:** 2018-03-16

**Authors:** Zsofia Miskolczi, Michael P. Smith, Emily J. Rowling, Jennifer Ferguson, Jorge Barriuso, Claudia Wellbrock

**Affiliations:** Manchester Cancer Research Centre, Faculty of Biology, Medicine and Health, School of Medical Sciences, Division of Cancer Sciences, The University of Manchester, Michael Smith Building, Oxford Road, Manchester, M13 9PT UK

## Abstract

Despite the general focus on an invasive and de-differentiated phenotype as main driver of cancer metastasis, in melanoma patients many metastatic lesions display a high degree of pigmentation, indicative for a differentiated phenotype. Indeed, studies in mice and fish show that melanoma cells switch to a differentiated phenotype at secondary sites, possibly because in melanoma differentiation is closely linked to proliferation through the lineage-specific transcriptional master regulator MITF. Importantly, while a lot of effort has gone into identifying factors that induce the de-differentiated/invasive phenotype, it is not well understood how the switch to the differentiated/proliferative phenotype is controlled. We identify collagen as a contributor to this switch. We demonstrate that collagen stiffness induces melanoma differentiation through a YAP/PAX3/MITF axis and show that in melanoma patients increased collagen abundance correlates with nuclear YAP localization. However, the interrogation of large patient datasets revealed that in the context of the tumour microenvironment, YAP function is more complex. In the absence of fibroblasts, YAP/PAX3-mediated transcription prevails, but in the presence of fibroblasts tumour growth factor-β suppresses YAP/PAX3-mediated MITF expression and induces YAP/TEAD/SMAD-driven transcription and a de-differentiated phenotype. Intriguingly, while high collagen expression is correlated with poorer patient survival, the worst prognosis is seen in patients with high collagen expression, who also express MITF target genes such as the differentiation markers *TRPM1*, *TYR* and *TYRP1*, as well as *CDK4*. In summary, we reveal a distinct lineage-specific route of YAP signalling that contributes to the regulation of melanoma pigmentation and uncovers a set of potential biomarkers predictive for poor survival.

## Introduction

Phenotypes impact on melanoma progression, and many in vitro studies and mouse models have concluded that it is the ‘de-differentiated/invasive’ phenotype that is more ‘aggressive’ and ‘metastatic’. Intriguingly, however, in patients many metastatic lesions display a high degree of pigmentation and as such differentiation. In fact, this should not be surprising, because in melanoma differentiation is closely linked to a ‘proliferative phenotype’ [1], and without proliferation there is no growth at distant sites. In line with this, in a zebrafish melanoma transplant model using a non-pigmented melanoma cell line, melanomas become differentiated after the dissemination to secondary sites [2]. Similar observations were made in mice injected with pigmented cells [3]. While the pigmented melanoma cells switched off this trait during early invasion and dissemination, the less differentiated phenotype was not maintained at secondary sites, and metastases were highly pigmented [3]. This suggests that differentiation was induced in these secondary tumours most probably by factors from the microenvironment.

The importance of such microenvironment-derived factors has been shown in the above-mentioned zebrafish transplant model, where depletion of EDN3, identified to induce both differentiation and proliferation, resulted in significant reduction of metastatic growth and increase in animal survival [2]. Despite the obvious relevance of proliferation for metastatic outgrowth, most studies so far have concentrated on identifying factors that induce the de-differentiated/invasive melanoma phenotype [4–6]. Much less is known with regard to factors that induce the differentiated/proliferative phenotype, and while apart from EDN3 several soluble factors are candidates for the induction of this phenotype, their presence most often depends on a tissue-specific microenvironment. On the other hand, a more general factor present in the tumour microenvironment that, however, has so far not been considered is the extracellular matrix (ECM).

Throughout melanoma development tumour cells are exposed to various types of ECM, with collagen being the most predominant matrix protein. Furthermore, melanoma progression is characterized by the increased presence of other matrix proteins such as tenascin-C and fibronectin [7], the latter affecting the organization of collagen fibres. Such structural changes in the matrix in addition to the expression of matrix proteases results in general matrix remodelling, and together this alters the mechanical properties of the ECM contributing to an overall more rigid tumour microenvironment [8]. Increasing evidence suggests that the tissue rigidity, or matrix stiffness controls phenotypic states and contributes to the acquisition of a malignant phenotype [9]. In epithelial cancers, a softer more compliant matrix enables differentiation, but a stiffer matrix can increase proliferation, induce EMT and transformation. In fact, high mammographic density linked to higher amounts of connective tissue is a marker for increased breast cancer risk [10].

At the molecular level integrins act as direct mediators between outside matrix signals and inside signalling via focal adhesion kinase (FAK) an SRC, but one of the main primary sensors of the physical nature of a cell and thus the mechanical properties of the ECM are the transcriptional co-activators YAP and its paralogue TAZ downstream of the actin cytoskeleton architecture and the Hippo signalling pathway [11]. In epithelial cancers, YAP/TAZ can initiate a cancer stem cell transcriptional programme, thus contributing to tumour initiation, progression and metastasis [12]. In melanoma, YAP and TAZ have been linked to a particular phenotype that is involved in resistance to targeted therapy [13, 14]. Furthermore, TEADs were identified as regulators of a phenotype that overlaps with the resistance as well as the de-differentiated/invasive phenotype [15]. The latter is in line with YAP and TAZ showing pro-invasive activity in melanoma, where they regulate the expression of the TEAD gene *CTGF* [16]. Thus, while there is some knowledge about the downstream activities of YAP and TAZ in melanoma, nothing is known about their function as ECM mechanosensors and how this regulates melanoma cell biology.

## Results

### Collagen stiffness regulates melanoma cell adhesion and nuclear YAP localization

**Fig. 1:**
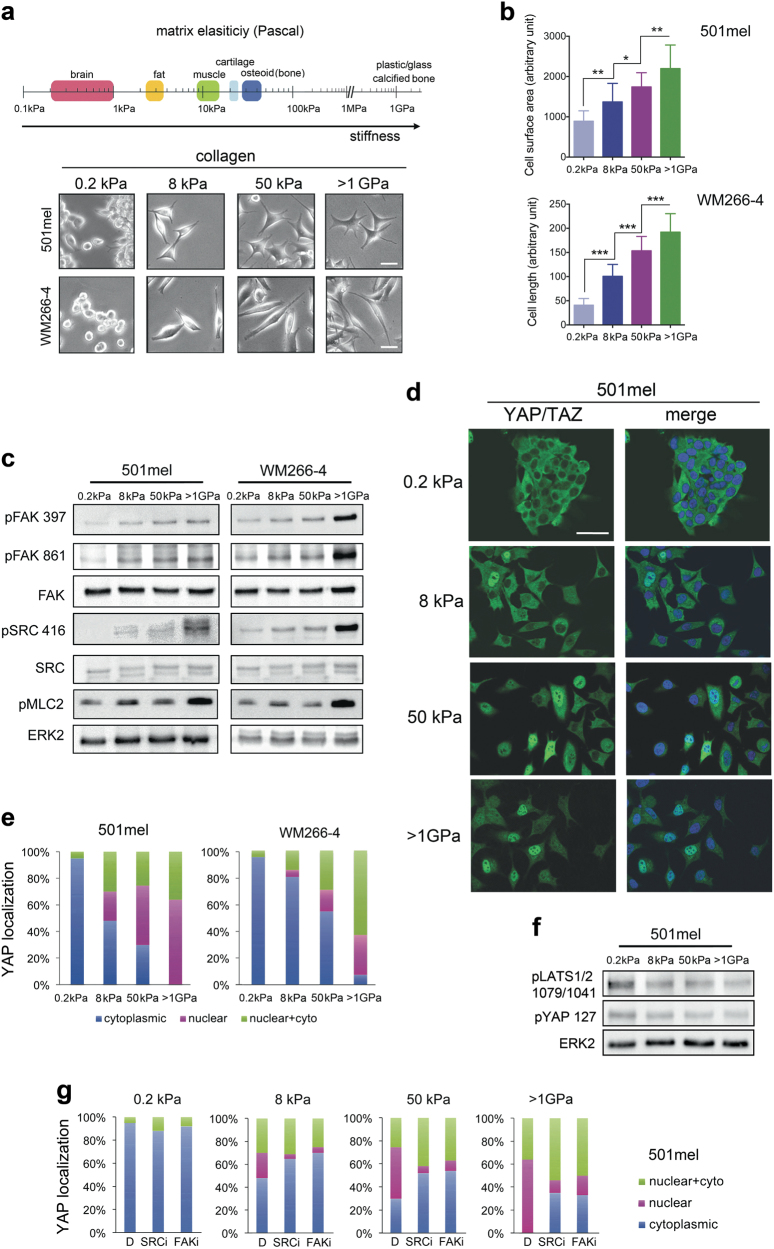
Mechanosignalling regulates melanoma cell adhesion and nuclear YAP/TAZ localization. **a** Morphology of 501mel and WM266-4 cells cultured for 72 h on collagen with the indicated stiffness degrees. A scale for matrix elasticity of tissues is shown varying from soft brain to rigid bone (adapted from [18]). Scale bar represents 20 µm. **b** Quantification of cell morphology of 501mel and WM266-4 cells cultured for 72 h on collagen with the indicated stiffness degrees (*n* = 3 experiments; *n* = 100 cells). Graphs show mean ± SEM. Tukey’s post-test following one-way ANOVA. *p < 0.05, **p < 0.01, ***p < 0.001. **c** Western blot analysis of pFAK, pSRC and pMLC2 of 501mel and WM266-4 cells cultured on collagen with the indicated stiffness degrees for 72 h. Total SRC, FAK and ERK2 was used as a loading control.**d** Immunofluorescence analysis of YAP/TAZ localization in 501mel cells cultured on collagen with the indicated stiffness degrees for 72 h. Scale bar represents 50 µm. **e** Quantification of YAP/TAZ localization in 501mel and WM266-4 cells cultured on collagen with the indicated stiffness degrees for 72 h (*n* = 3 experiments; *n* = 100 cells). **f** Western blot for LATS and YAP phosphorylation in 501mel cells cultured on collagen with the indicated stiffness degrees for 72 h. **g** Quantification of YAP/TAZ localization in 501mel cells cultured on collagen with the indicated stiffness degrees treated with 1 μM AZD0530 (SRCi), 1 μM PF562271 (FAKi) or DMSO for 24 h (*n* = 3 experiments; *n* = 100 cells)

In order to assess the influence of differing mechanical properties of collagen on the behaviour of melanoma cells we used collagen-coated hydrogels. This allowed us to expose cells to collagen of varying stiffness, such as 0.2 and 8 kPa resembling brain and skin, respectively, or 50 kPa, representing a slightly stiffer environment similar to what is found in tumours [17, 18]. Collagen-coated tissue culture plates (>1 GPa) are more representative for calcified bone (Fig. 1a, schematic adapted from [18]).

Melanoma cells plated on these various collagen gels showed a different morphology, more rounded and less adherent on a 0.2 kPa gel and more spread with increasing stiffness (Fig. 1a). This was quantified in WM266-4 cells as cell length and in 501mel cells as cell area (Fig. 1b). In line with greater spreading to a stiffer matrix, we saw a rise in vinculin-containing focal adhesions (Supplementary Figure S1a), and increased SRC and FAK phosphorylation (Fig. 1c). Furthermore MLC2 phosphorylation was elevated (Fig. 1c), possibly due to higher numbers of RHO-dependent stress fibres found in cells on stiff matrix [19]. YAP/TAZ, major mechanosensors downstream of SRC, FAK and RHO [19, 20], showed more nuclear localization in cells plated on stiffer collagen (Fig. 1d and e; Supplementary Figures S1b and c). This correlated with gradual changes in LATS and YAP phosphorylation (Fig. 1f) and was dependent on active SRC, FAK and ROCK (Fig. 1g and Supplementary Figures S2a–d). Finally, affecting focal adhesions resulted in reduced YAP expression (Supplementary Figure S2e), further emphasizing the close link of this mechanosensor to cell adhesion.

### Nuclear YAP/TAZ expression correlates with increased collagen abundance in melanoma

**Fig. 2:**
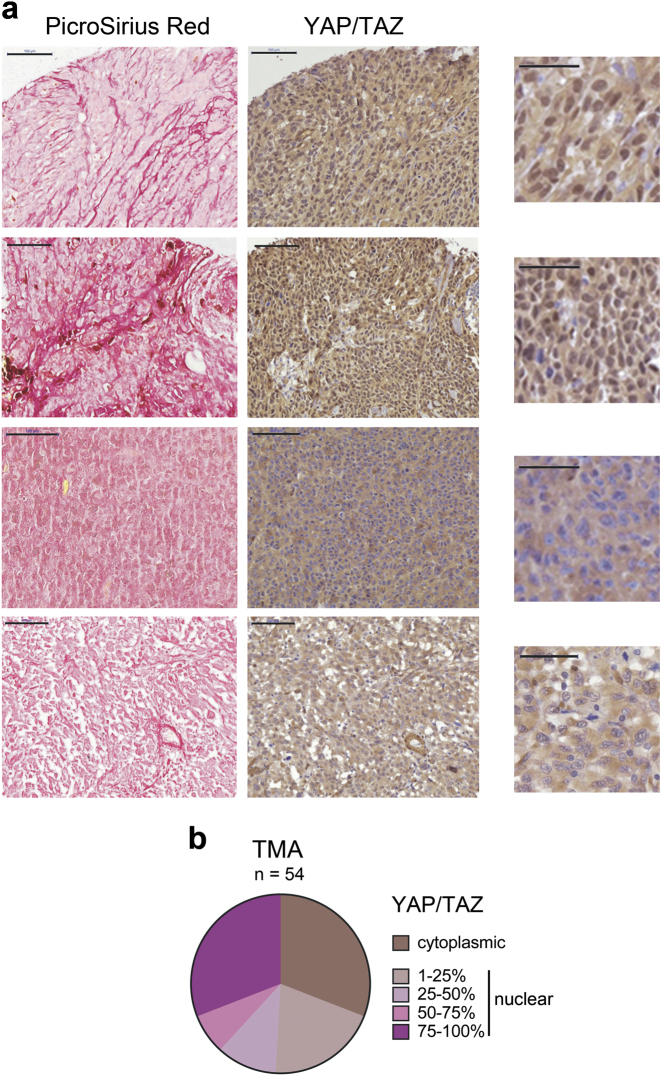
Increased collagen deposition correlates with nuclear YAP/TAZ localization. **a** Immunohistochemistry of a tissue microarray of primary and metastatic melanocytic lesions stained with PicroSirius Red (for collagen) and a YAP/TAZ-specific antibody. Scale bars represent 100 μm, and 50 µm for the magnifications. **b** Quantification of YAP/TAZ localization in the nucleus/cytoplasm of tissue microarray of primary and metastatic melanocytic lesions (*n* = 54)

Tumours are often stiffer than healthy tissue and for melanoma changes in mechanical properties are seen as early as in melanocytic nevi [21]. This stiffness is partly attributed to increased matrix deposition and reorganization involving collagen and fibronectin. In order to assess whether there is a correlation with increased collagen and YAP localization, we analysed a tissue microarray (TMA) containing primary as well as metastatic melanocytic lesions (Table S1). We stained the samples for expression of collagen (PicroSirius Red), fibronectin and YAP/TAZ (Fig. 2a and Supplementary Figure S3a).

We could identify tumours with high and low collagen abundance (Fig. 2a). In most cases, increased fibronectin expression was found in tumours with high collagen abundance (Supplementary Figure S3a), and cytoplasmic YAP/TAZ was distinguishable from nuclear YAP/TAZ (Fig. 2a). Quantification revealed that approximately two-thirds (37/54) of all analysed samples displayed nuclear YAP/TAZ to various degrees, whereas in one-third (18/54) of the samples YAP/TAZ was cytoplasmic (Fig. 2b). Intriguingly, there was a weak, but significant correlation (Pearson's correlation, *P* = 0.0232) of nuclear YAP localization with overall matrix abundance (Supplementary Figure S3b).

### YAP regulates MITF expression in melanoma

**Fig. 3:**
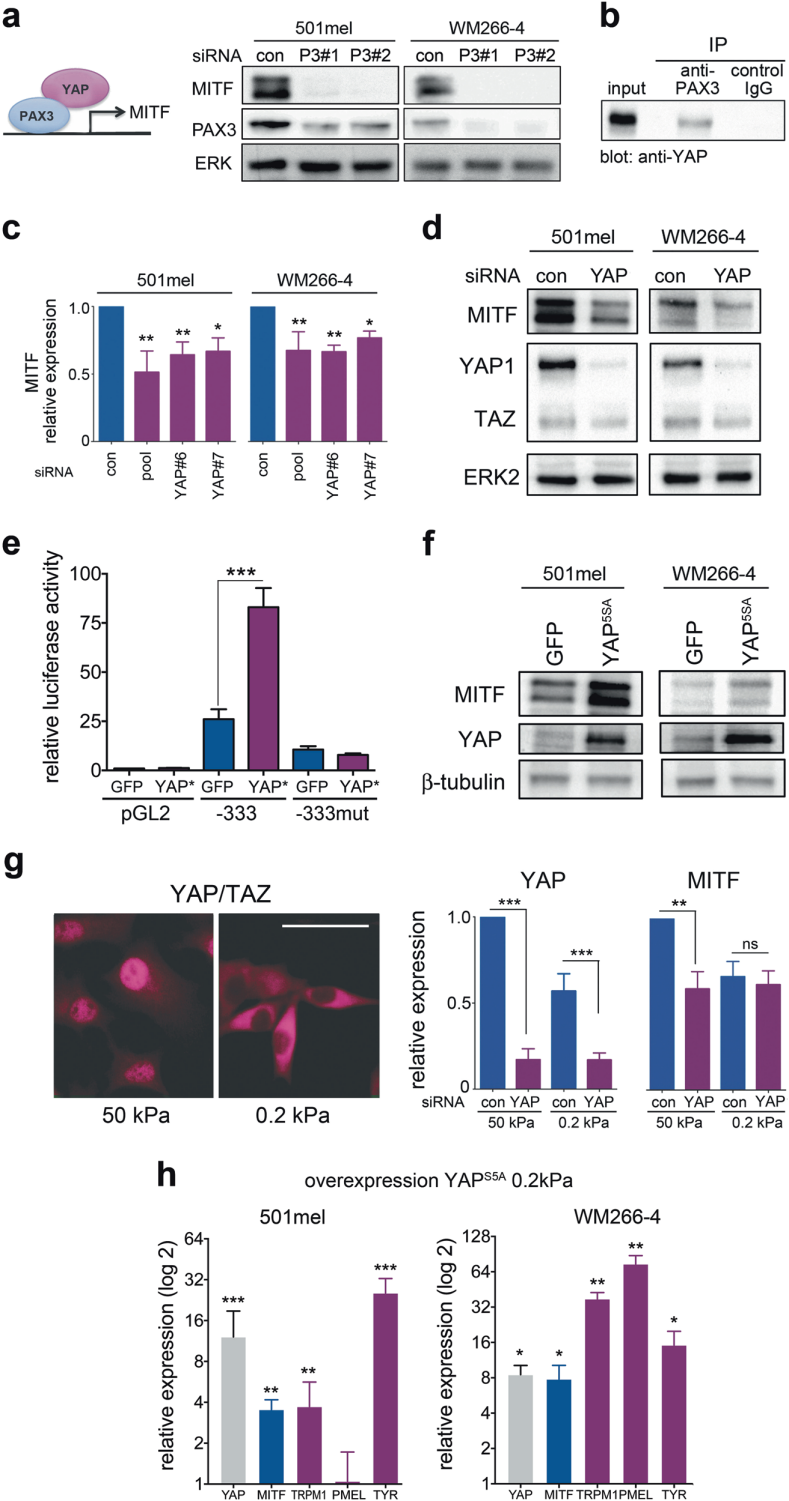
YAP regulates MITF expression in melanoma. **a** Western blot analysis of MITF and PAX3 of 501mel and WM266-4 cells treated with PAX3-specific siRNAs (#1 and #2) for 48 h. ERK2 served as a loading control. A model indicating the PAX3-YAP interaction at the *MITF* promoter is shown. **b** Co-Immunoprecipitation of YAP and PAX3 using an anti-PAX3 antibody for precipitation and an anti-YAP antibody for Western blot analysis of the precipitate. **c** qRT-PCR analysis of MITF expression in 501mel and WM266-4 cells upon treatment with three YAP-specific siRNAs (ON-TARGET*plus SMART*pool, #6 and #7) for 48 h. Graphs show mean ± SEM. Tukey’s post-test following one-way ANOVA. n.s., not significant, *p < 0.05, **p < 0.01. **d** Western blot analysis of MITF and YAP/TAZ in 501mel and WM266-4 cells upon YAP siRNA (SMARTpool) treatment for 48 h. ERK2 served as a loading control. **e** Luciferase reporter assay for transcriptional activity driven from the indicated *MITF* promoter fragments in the presence of over expressed GFP or YAP^5SA^ in 501mel cells. **f** Western blot analysis of MITF and YAP of 501mel and WM266-4 cells upon overexpression of YAP5SA. β-tubulin served as a loading control. **g** Immunofluorescence analysis of YAP/TAZ of 501mel cells and qRT-PCR analysis of MITF and YAP expression in 501mel cells upon YAP siRNA (ON-TARGETplus SMARTpool) treatment cultured on 50 and 0.2 kPa collagen gels. Graphs show mean ± SEM. Tukey’s post-test following one-way ANOVA. n.s., not significant, **p < 0.01, ***p < 0.001. Scale bar represents 50 µm. **h** qRT-PCR analysis for the expression of *YAP*, *MITF* and the MITF target genes *TRPM1*, *PMEL* and *TYR* in 501mel and WM266-4 cells cultured on a 0.2 kPa collagen gel upon overexpression of YAP5SA. Graphs show mean ± SEM. Tukey’s post-test following one-way ANOVA. n.s., not significant, *p < 0.05, **p < 0.01

Next, we assessed the molecular consequences of increased nuclear YAP/TAZ in melanomas. In mice Yap/Taz drive the commitment of the melanocyte lineage by acting as co-activators of Pax transcription factors in the neural crest [22]. In this context, a Yap/Pax3 complex induces the expression of the melanocyte lineage commitment factor Mitf. This is most intriguing, as MITF also plays an important role in melanoma, where it is a central regulator of melanoma cell survival, proliferation and differentiation, and as such, considered the marker of the ‘differentiated/proliferative’ phenotype [1, 23]. Furthermore, MITF expression is regulated by PAX3, whose expression is controlled downstream of oncogenic BRAF and the mitogen-activated protein kinase pathway [24]. We could confirm the crucial role of PAX3 in the regulation of MITF expression in both 501mel and WM266-4 cells (Fig. 3a). We found that PAX3 and YAP interact in melanoma cells (Fig. 3b) and that depletion of YAP using small interfering RNA (siRNAs) results in decreased MITF mRNA expression in cells cultured on >1 GPa collagen (Fig. 3c). Decreased MITF expression after YAP depletion was also observed at the protein level (Fig. 3d). Furthermore, the YAP mutant YAP5SA that displays constitutive nuclear localization [25] could induce expression from a ~300 bp fragment of the *M-MITF* promoter [26], but only when the PAX3 binding site was intact (Fig. 3e), indicating that efficient PAX3 activity is required for YAP-induced expression of MITF. Finally, overexpression of YAP5SA in also induced increased expression of endogenous MITF (Fig. 3f). Together, these data indicate that YAP regulates MITF expression in melanoma cells in a PAX3-dependent manner.

The relevance of the YAP-mediated MITF expression in the context of matrix stiffness was further confirmed using RNA interference. On a stiff matrix when YAP is nuclear and co-localizes with PAX3 (Supplementary Figure S4), YAP depletion resulted in a significant decrease in MITF expression (Fig. 3g), but there was no contribution of YAP to MITF expression in melanoma cells grown on a 0.2 kPa soft matrix when the majority of YAP is cytoplasmic (Fig. 3g). However, overexpression of ‘nuclear’ YAPS5A-induced expression of MITF and its target genes also on soft matrix (Fig. 3h).

### Increased collagen stiffness induces proliferation and differentiation in melanoma cells

**Fig. 4:**
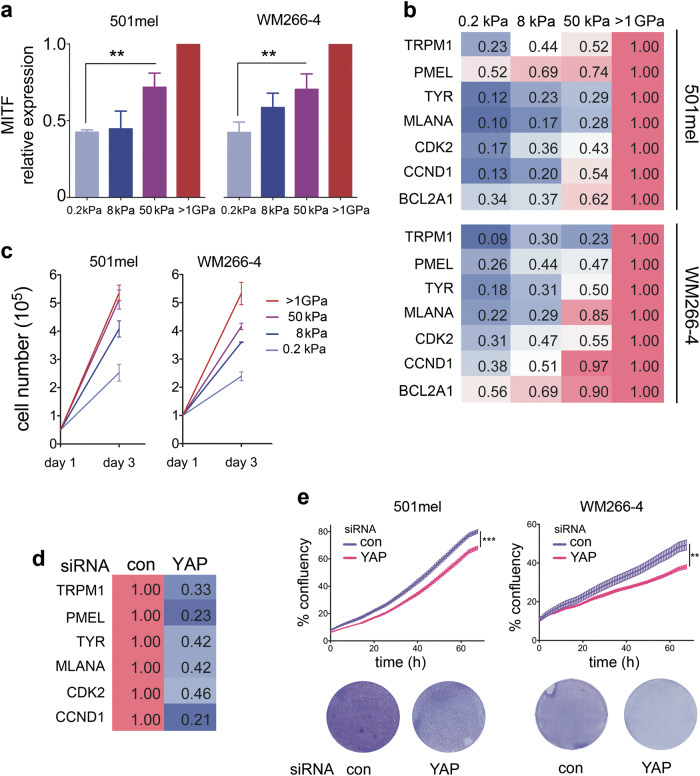
Increased collagen stiffness promotes proliferation and differentiation via YAP1/MITF. **a** qRT-PCR analysis of MITF expression in 501mel and WM266-4 cells cultured on collagen with the indicated stiffness degrees for 7 days. Graphs show mean ± SEM. Tukey’s post-test following one-way ANOVA. **p < 0.01. **b** Heatmap of relative expression of differentiation (*TRPM1*, *PMEL*, *TYR* and *MLANA*), proliferation (*CDK2* and *CCND1*) and survival (*BCL2A1*) genes in 501mel and WM266-4 cells cultured on collagen with the indicated stiffness degrees for 7 days. **c** Quantification of proliferation of 501mel and WM266-4 cells cultured on collagen with the indicated stiffness degrees for 72 h. **d** Heatmap of relative expression of differentiation (*TRPM1*, *PMEL*, *TYR* and *MLANA*) and proliferation genes (*CDK2* and *CCND1*) in 501mel cells upon YAP siRNA (ON-TARGETplus SMARTpool) treatment. **e** Incucyte analysis and crystal violet staining to measure cell growth (confluency) of 501mel and WM266-4 cells upon YAP siRNA (SMARTpool) treatment

As a consequence of YAP’s contribution to MITF regulation, we expected an impact of matrix stiffness on MITF mRNA levels, and indeed we found a gradual increase of MITF expression with increased collagen stiffness (Fig. 4a). Because MITF is a central regulator of melanoma biology, we analysed the expression of crucial MITF ‘signature’ target genes, which revealed that the relative expression of melanoma differentiation genes (*TRPM1*, *PMEL*, *TYR* and *MLANA*) as well as proliferation and survival genes (*CDK2*, *BCL2A1*) followed MITF expression and was higher in stiffer matrix (Fig. 4b). Indicative for a ‘proliferative’ phenotype, *CCND1* expression was also up-regulated and the melanoma cell numbers increased more over 3 days on stiffer matrix (Fig. 4c). In line with YAP regulating MITF expression, we found that YAP depletion resulted in the reduced expression of MITF target genes, including not only differentiation but also proliferation markers (Fig. 4d), and indeed, melanoma cell proliferation was significantly reduced after YAP knockdown (Fig. 4e and Supplementary Figure S5).

### Fibroblast markers inversely correlate with the ‘high-collagen’ proliferation and differentiation state

**Fig. 5:**
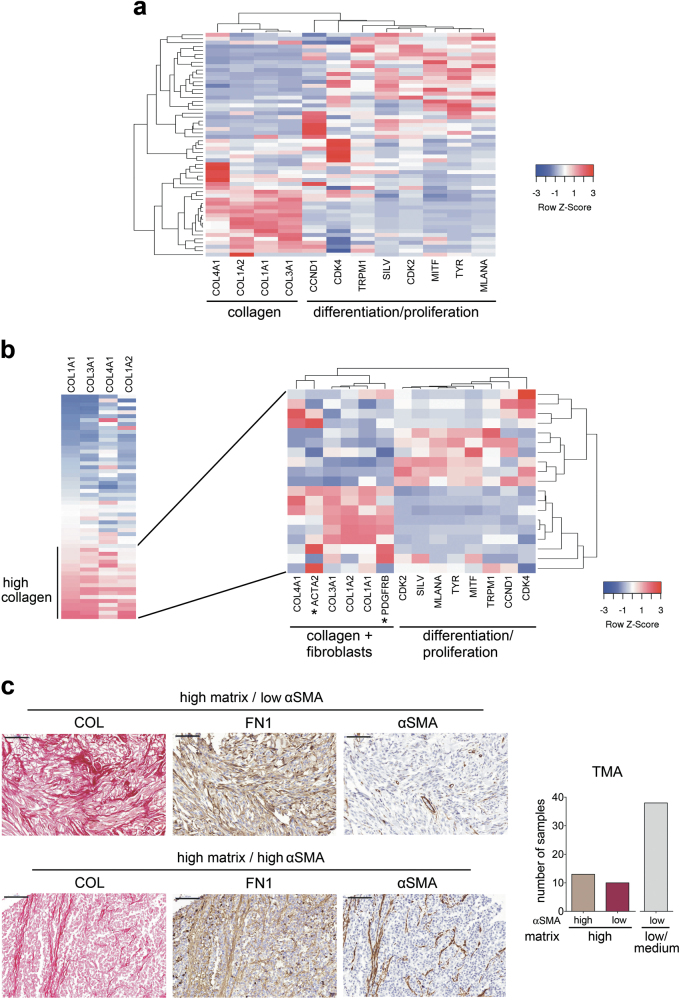
Fibroblast infiltration inversely correlates with a proliferation/differentiation state. **a** Hierarchical clustering of gene expression data from a cohort of 57 stage IV melanomas using the Jonsson dataset (GEO number: GSE22153 and see [27]). **b** Hierarchical clustering of gene expression data in a ‘high collagen’ expression cohort using the Jonsson dataset (GEO number: GSE22153). Patients with high expression of collagen type I were selected and re-analysed including fibroblasts markers *ACTA2* and *PDGFRB* (indicated by *). **c** Immunohistochemistry of a tissue microarray of primary and metastatic melanocytic lesions stained with PicroSirius Red (for collagen), fibronectin (FN1) and αSMA (*ACTA2*, fibroblast marker) antibodies and quantification of matrix (FN1 + COL) and αSMA staining intensity. Scale bars represent 100 μm

Our results reveal a link between collagen abundance, nuclear YAP localisation and MITF target gene expression. To further validate this link, we interrogated an expression dataset from 57 stage IV melanomas [27]. Unsupervised hierarchical clustering revealed a mutual exclusive expression of collagen genes and MITF signature genes (Fig. 5a). While this is in line with data from the previous analysis [27], it contradicted our observation that high collagen abundance in melanocytic lesions correlates with nuclear YAP, which we find induces MITF expression, and consequently a differentiation/proliferation signature.

We therefore revisited our analysis, and focused on the ‘high collagen’ expression cohort, which represents approximately one-third of the cohort (19 patients). We then considered that the source of the collagen within a tumour could vary; we have previously shown that collagen deposition in vivo can be performed by melanoma cells themselves [28], but it is well known that resident fibroblasts can also contribute to matrix deposition [29]. Considering the presence of markers for tumour-associated fibroblasts such as αSMA (ACTA) and PDGFRB, we found that the ‘high collagen’ dataset segregated into groups with high and low MITF ‘signature’ gene expression, and this was inversely correlated with the expression of the fibroblast markers *ACTA2* and *PDGFRB* (Fig. 5b). This suggested that melanomas divide into groups of tumours with high and low fibroblast infiltration, and indeed when we stained our TMA samples for αSMA, we found that approximately 50% of the high-matrix samples was positive for the presence of the fibroblast marker αSMA (Fig. 5c).

### Melanoma cells stimulate fibroblast-mediated matrix deposition and remodelling

**Fig. 6:**
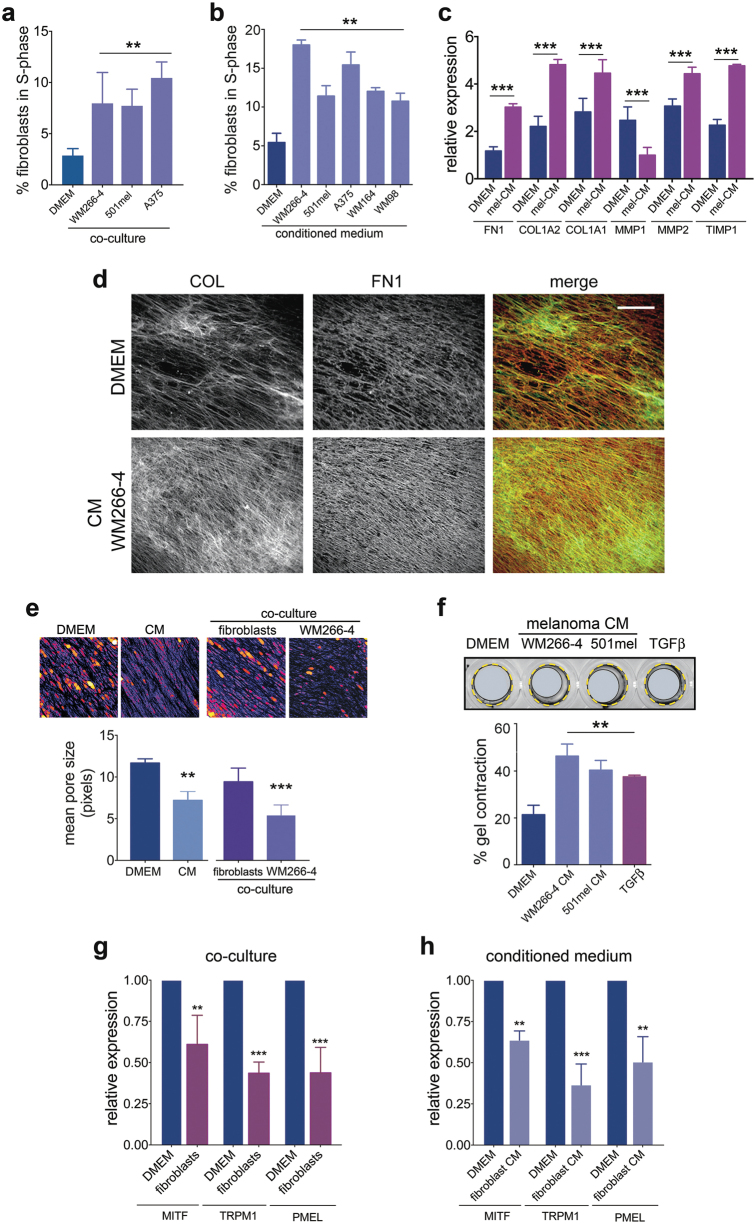
Melanoma cells stimulate fibroblast-mediated matrix deposition and remodelling. **a** Quantification of fibroblast proliferation using Edu incorporation after co-culture with the indicated melanoma cell lines for 7 days. Graphs show mean ± SEM. Tukey’s post-test following one-way ANOVA. **p < 0.01. **b** Quantification of fibroblast proliferation using Edu incorporation after treatment with conditioned media from a panel of melanoma cell lines for 7 days. Graphs show mean ± SEM. Tukey’s post-test following one-way ANOVA. **p < 0.01. **c** qRT-PCR analysis of expression of matrix genes (*FN1*, *COL1A1* and *COL1A2*) and matrix remodelling genes (*MMP1*, *MMP2* and *TIMP1*) in fibroblasts treated with WM266-4 conditioned medium (mel-CM) for 7 days. Graphs show mean ± SEM. Tukey’s post-test following one-way ANOVA. **p < 0.01, ***p < 0.001. **d **Immunofluorescence analysis of cell-derived matrix from fibroblasts treated with WM266-4 conditioned medium, using collagen-specific (COL1) and fibronectin-specific (FN1) antibodies. Scale bar represents 100 µm. **e** Pore size analysis of cell-derived matrix from fibroblasts treated with WM266-4 CM or co-cultured with WM266-4 cells. Images generated by immunofluorescence (example shown) were binary transferred and pore size was measured by using BoneJ plugin Thickness/Space function. Quantification of mean pore size of images is shown (*n* = 3 experiments; *n* = 10 images). **f** Gel contraction assay of fibroblasts embedded in collagen gel and treated with conditioned media from 501mel and WM266-4 cells or with 20 ng/ml TGFβ as a positive control. The perimeter of the collagen gel was measured after 72 h. Graphs show mean ± SEM. Tukey’s post-test following one-way ANOVA. **p < 0.01. **g** qRT-PCR analysis of *MITF*, *TRPM1* and *PMEL* expression in WM266-4 cells co-cultured with fibroblast for 7 days. Graphs show mean ± SEM. Tukey’s post-test following one-way ANOVA. **p < 0.01, ***p < 0.001. **h** qRT-PCR analysis of *MITF*, *TRPM1* and *PMEL* expression in WM266-4 cells treated with fibroblast conditioned medium (fibroblast-CM) for 7 days. Graphs show mean ± SEM. Tukey’s post-test following one-way ANOVA. **p < 0.01, ***p < 0.001

Because the presence of fibroblasts impacted on the MITF signature and as such the melanoma phenotype, we further analysed the communication between melanoma cells and fibroblasts. Co-culture of melanoma cells with fibroblasts resulted in increased fibroblast proliferation, and similar effects were seen with conditioned medium (CM) from a broader panel of melanoma cell lines (Fig. 6a, b).

Melanoma-CM also influenced the expression of matrix genes as well as genes encoding proteins that impact on matrix remodelling, such as *MMP2* and *TIMP1* (Fig. 6c). Indeed, when we analysed the collagen and fibronectin deposited by fibroblasts, we found that melanoma-CM or co-culture with melanoma cells increased matrix abundance and the overall matrix density determined by reduced pore size (Fig. 6d, e). In addition, melanoma-CM activates fibroblasts to contract matrix (Fig. 6f). These data suggest that melanoma cells impact on fibroblasts to produce a denser and stiffer matrix, and in line with this, melanoma cells cultured on isolated fibroblast-derived matrix were more proliferative (not shown). However, our expression data analysis (see Fig. 5b) indicated that the presence of fibroblasts is correlated with reduced MITF expression. This suggested that other fibroblast-derived factors than the ECM dominate signalling to reduce MITF expression and the differentiation/proliferation phenotype. Indeed, we found that co-culture with fibroblasts resulted in reduced expression of MITF and its target genes in melanoma cells (Fig. 6g). A similar effect was seen with fibroblast-CM (Fig. 6h), indicating that fibroblasts secrete factors that reduce MITF activity.

### Differential YAP-mediated transcription impacts on MITF target gene expression

**Fig. 7:**
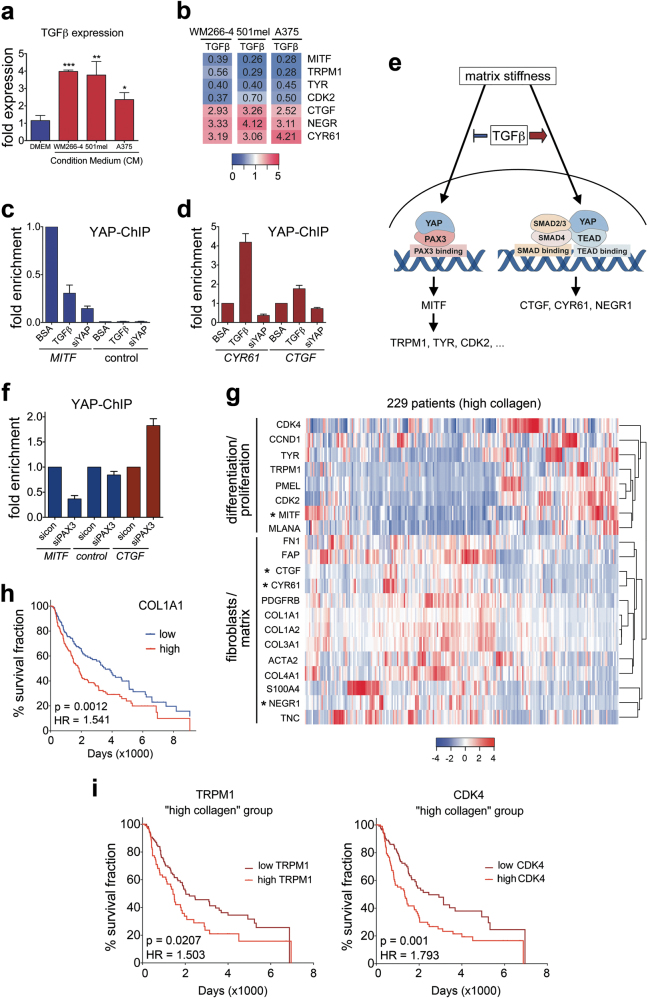
Differential YAP-meditated transcription is influenced by fibroblast infiltration and impacts on patient survival. **a** qRT-PCR analysis of expression of TGFβ in fibroblasts treated with WM266-4, 501mel or A375 conditioned media for 7 days. Graphs show mean ± SEM. Tukey’s post-test following one-way ANOVA. *p < 0.05, **p < 0.01, ***p < 0.001. **b** Heatmap of relative expression of MITF and its target genes (*TRPM1*, *TYR* and *CDK2*) and YAP/TEAD target genes (*CTGF*, *NEGR* and *CYR61*) in WM266-4, 501mel and A375 cells cultured on >1 GPa after treatment with 20 ng/ml TGFβ for 24 h. The expression of the respective genes in untreated cells was set 1 (see also colour key). **c** ChIP-PCR analysis in 501mel cells to assess enrichment of YAP at the *MITF* promoter of the following 20 ng/ml TGFβ treatment or YAP depletion. BSA-treated cells were used as baseline YAP-binding activity, and amplification of an intronic sequence of the *MITF* gene was used as a negative control. **d** ChIP-PCR analysis in 501mel cells to assess enrichment of YAP at the promoters of *CYR61* and *CTGF* following 20 ng/ml TGFβ treatment or YAP depletion. BSA-treated cells were used as baseline YAP-binding activity. **e** Schematic of YAP-driven transcription in the presence or absence of TGFβ in the microenvironment. When TGFβ signalling is active, YAP interacts with TEAD/SMADs to drive the expression of *CTGF*, *CYR61* and *NEGR1* and inhibit YAP/PAX3-mediated transcription of *MITF*. **f** ChIP-PCR analysis in 501mel cells to assess enrichment of YAP at the promoters of *MITF* and *CTGF* following PAX3 depletion by RNAi. BSA-treated cells were used as baseline YAP-binding activity and amplification of an intronic sequence of the *MITF* gene was used as a negative control. Binding in the presence of a control siRNA was set 1. **g** Hierarchical clustering of gene expression data derived from the TCGA melanoma dataset stratified for ‘high collagen’ expression (229 patients, upper 50%). **h** Kaplan–Meier analysis using the TCGA melanoma dataset. Differences in overall survival in two cohorts (upper and lower 50%) with low and high COL1A1 expression are shown. *n* = 229 per cohort; Hazard ratio (HR) log-rank = 1.575 for high collagen (95% CI: 1.205–2.057); *p* (log-rank) = 0.0007. **i** Kaplan–Meier analyses using the TCGA melanoma dataset. Differences in overall survival for high and low expression of *TRPM1* or *CDK4*, respectively, in the ‘high collagen’ group (*n* = 229) are shown. For *TRPM1*: hazard ratio (HR) log-rank 1.61 for high TRPM1 (95% CI: 1.107–2.36); *p* (log-rank) = 0.0073; for *CDK4*: hazard ratio (HR) log-rank 1.706 for high CDK4 (95% CI : 1.189–2.448); *p* (log-rank) = 0.0028

One fibroblast-secreted factor is tumour growth factor-β (TGFβ), whose production was increased in the presence of melanoma-CM (Fig. 7a). In agreement with previous reports [30–32], TGFβ suppressed the expression of MITF and its target genes (Fig. 7b). On the other hand, TGFβ induced the expression of *CTGF*, *NEGR1* and *CYR61* (Fig. 7b). Importantly, in contrast to MITF as YAP/PAX3 target gene [22], these TGFβ-induced genes are regulated by SMADs in a complex with YAP and TEAD [33–35]. This suggests that in melanoma cells TGFβ can trigger a switch from YAP/PAX3-driven transcription to SMAD/YAP/TEAD-driven transcription, an idea that is further supported by the fact that TGFβ suppresses PAX3 expression [31, 32], thus reducing the amount of PAX3 available for YAP binding.

To test our hypothesis of differential YAP-mediated transcription, we performed YAP-chromatin immunoprecipitation (ChIP), which identified YAP at the *MITF* promoter (Fig. 7c) at a previously identified binding site [22]. The binding was specific, as it was blocked when YAP was depleted (Fig. 7c). Also, TGFβ reduced the amount of YAP at the *MITF* promoter (Fig. 7c). The situation was quite different at the promoters of *CTGF* and *CYR61*, where YAP binding increased in response to TGFβ (Fig. 7d). Together, this leaves us with a model in which YAP can interact with PAX3 to drive expression from the *MITF* promoter, contributing to an overall differentiated and proliferative phenotype. However, the presence of TGFβ induces a switch from YAP/PAX3 to a SMAD-driven and YAP/TEAD-driven transcription programme, which establishes a de-differentiated slow cycling phenotype (Fig. 7e). The key role of PAX3 in this switch model is confirmed by the fact that its depletion resulted in reduced YAP binding at the *MITF* promoter, but increased YAP at the *CTGF* promoter (Fig. 7f).

### MITF target gene expression correlates with poor survival in the high-collagen cohort

To further validate the differential regulation of YAP target genes in melanoma patients, we interrogated the TCGA melanoma dataset, which contains expression data from 458 patients of various stages of melanoma [36]. Again, hierarchical clustering of the complete dataset identified mutual exclusion of collagen genes and MITF target genes (Figure S6). When we concentrated on the ‘high collagen’ expression cohort, we found the same two groups as seen previously: COL-high/MITF-high and COL-high/MITF-low. Again, genes clustering with high MITF were linked to differentiation and proliferation, but fibroblast marker genes such as *ACTA2*, *PDGFRB*,* FAP and S100A4* associated with low MITF expression (Fig. 7g). Supporting our model, the TGFβ/SMAD/TEAD-regulated genes *CTGF*, *CYR61* and *NEGR1* clustered with the fibroblast markers.

Because matrix abundance and fibroblasts impact on melanoma phenotypes, we wanted to investigate the clinical relevance of these parameters. We first analysed the effect of matrix abundance on patient survival. In the TCGA patient cohort high levels of COL1A1, COL3A1 and COL4A1 were all associated with reduced survival (Fig. 7h and Supplementary Figure S7). Patients with high COL1A1 expression had a median survival of 61 months compared to 117 months for patients with low COL1A1 expression (Fig. 7h). Most intriguingly, however, when we focused on the high COL1A1 cohort and further stratified the data according to their differentiation and proliferation status by looking at *TRPM1*, *TYRP1* and *CDK4*, the median survival was even lower and dropped below 50 months (Fig. 7i), clearly emphasizing that the differentiation state of melanomas could act as prognostic factor.

## Discussion

The ECM plays an important role in normal physiological conditions and altered ECM deposition and degradation is associated with many diseases, including cancer. We found that high amounts of collagen, as well as fibronectin (FN1) were present in approximately 30–40% of melanomas, with another 30–40% containing moderate amounts of matrix. A similar observation had been made using antibodies specific to individual domains of FN1, which also detected the occurrence of matrix remodelling during melanoma progression [7]. Indeed tumours generally display increased abundance and remodelled matrix, which in turn impacts on the mechanical properties of malignant lesions. This phenomenon is well studied in breast cancer, where collagen cross-linking and matrix stiffening induces enhanced integrin signalling and stimulates the invasion of an oncogene-initiated epithelium [37]. While pigmented lesions such as nevi as well as melanomas also display greater stiffness than normal skin [21], little is known about how mechanical properties of the matrix impact on melanoma initiation or progression. On the other hand, there is a well-documented link between altered ECM and skin pigmentation. For instance, excess fibrotic connective tissue, which is stiffer than healthy skin, is frequently correlated with hyper-pigmentation [38], and in scar tissue that contains altered ECM as the result of wound healing, both hyper-pigmentation and hypo-pigmentation can be observed [39]. This indicates that not only the ECM can impact on the differentiation state of melanocytes but also that this is a complex situation where opposite outcomes can be established.

Our findings suggest that the opposite outcomes described above might reflect the individual involvement of fibroblasts, which are recruited and become activated during the normal wound-healing process. Also, cancer cells attract and activate fibroblasts leading to the dense, fibrotic appearance of tumours through ECM deposition. Although the role of these cancer-associated fibroblasts (CAFs) has mostly been studied in epithelial-derived tumours, there is increasing evidence for the importance of CAFs in melanoma. Melanoma cells secrete a cocktail of factors including TGFβ, platelet-derived growth factor, basic fibroblast growth factor and tumour necrosis factor to stimulate fibroblast activity [29, 40]. In return, CAFs signal to melanoma cells to support tumour growth. In vitro fibroblasts are recruited to melanoma spheroids within days, and this results in overall increased matrix abundance within the spheroid [41]. However, we have shown previously that in vivo, apart from fibroblasts, melanoma cells are also capable of producing a functional matrix including collagen and fibronectin [28]. This is in line with our observation that not all melanomas with high matrix abundance stained also positive for αSMA. In fact, only 56% of melanomas with high matrix abundance showed strong αSMA staining. A similar trend was reflected in the gene expression data in both the Jonsson and the TCGA dataset.

We have demonstrated that collagen impacts on melanoma phenotypes in vitro, whereby high collagen abundance/stiffness stimulates a differentiated/proliferative phenotype, but soft matrix induces a slow cycling more de-differentiated phenotype. Nevertheless, in vivo the presence of fibroblasts creates a more complex situation, because once recruited, melanoma cells can stimulate fibroblasts to express a number of growth factors and cytokines, including TGFβ [40, 42]. TGFβ plays a crucial role in the melanocyte lineage, where it can maintain the ‘melanocyte stem cell state’ by blocking MITF expression [30]. In differentiated melanocytes, TGFβ can reduce MITF through the suppression of PAX3 [32], and we have recently shown that this regulatory link is preserved in melanoma cells [31]. This finding is entirely in line with the observation that TGFβ can suppress pigmentation in melanoma cells [3].

Intriguingly, the transcriptional state linked to the ‘melanocyte stem cell state’ maintained by TGFβ is similar to what was identified as a MITF-low ‘TEAD-regulated invasive state’ [15], and our data suggest that TGFβ induces gene expression partly through integrating a YAP/TEAD complex into SMAD directed transcription. Because TGFβ signalling is linked to tumour progression, YAP has been linked to cancer progression in the context of TEAD-driven expression, and also in melanoma YAP can promote metastasis in a TEAD-dependent manner [43]. However, we discovered a novel role for YAP in melanoma, where it acts as cofactor for PAX3-driven expression of MITF, thereby regulation not only proliferation but also differentiation. Advanced melanomas are frequently pigmented indicating that differentiation does not impede metastatic growth, most probably because in melanoma the differentiated phenotype is closely linked to proliferation through MITF. Indeed, time-lapse imaging in zebrafish has revealed that differentiated/pigmented melanocytes are capable of cell division [44]. Moreover, MITF has been shown to be required for metastatic growth in mice, where depletion of MITF reduced the number and area of lung metastases [45].

Our survival analyses in patients reveal that a high degree of differentiation is correlated with significantly shorter median survival. Indeed, the correlation of reduced survival with increased differentiation/pigmentation in melanoma has been observed previously [27, 46–48]. Moreover, it has been reported that melanin levels measured in primary melanomas developing metastases are higher than in the ones that do not develop metastasis [46]. As pigmented melanomas are of the MITF-high phenotype, the latter is challenging the general belief that it is the MITF-low cell population that is most ‘metastatic’. In this context, Jonsson and co-workers have identified a melanoma ‘high-immune signature’, which is significantly correlated with better survival and which intriguingly is characterized by low MITF expression [27]. Thus, the situation appears to be more complex and simply dividing ‘aggressive’ and ‘non-aggressive’ melanomas by their MITF expression state is not sufficient. Our data demonstrate that additional factors that need to be considered are coming from the tumour microenvironment and involve not only fibroblasts as a cellular component but also the abundance and properties of the ECM.

## Materials and methods

### Cell culture and cell-derived matrix

A375 and WM266-4 cells were from ATCC. 501mel cells were a gift from Steven Rosenberg (NCI, Bethesda, MD, USA). Cells have been authenticated in-house in 2017, were cultured in Dulbecco's modified Eagle's medium (DMEM)/10% fetal calf serum (FCS)/0.5% pen-strep (PAA, Yeovil, UK) and mycoplasma-tested. Human dermal fibroblasts were a gift from Guillaume Jacquement (Manchester) and were grown in DMEM/5% FCS. Co-cultures were set up as described previously [49]. Melanoma-CM was taken from confluent flasks of melanoma cells grown in DMEM/10% FCS and filtered (0.45 μm). Cell-derived matrix was generated as previously described [50].

### Transfection, siRNAs and drugs

WM266-4 cells were transfected with different siRNAs using INTERFERin (Polyplus, Illkirch, France). 501mel and A375 cells were transfected using Lipofectamine^®^ (Life Technologies) following the protocol described by the manufacturer. siRNA sequences were as follows: scrambled control, 5′-AAUAUAAUCACUAUCAGGUGC-3′; YAP1#06, 5′-UGAGAACAAUGACGACCAA-3′; YAP1#07, 5′-GGUCAGAGAUACUUCUUAA-3′; YAP1 pool (Dharmacon, L-012200-00-0005 ON-TARGET*plus* siRNA-*SMART*pool); PAX3#1, 5′-CCGAGACAAAUUACUCAAGGA-3′; PAX#2, 5′-GAAACACCGUGCCGUCAGUUU-3′. AZD0530 (SRCi) and PF562271 (FAKi) were from Selleckchem (Newmarket, UK).

### EdU incorporation and IncuCyte analysis

Cells were labelled with 20 µM 5-ethynyl-2'-deoxyuridine (EdU) for 4–24 h and processed following the manufacturer’s protocol (Click-iT^®^ EdU Alexa Fluor^®^ 488 Imaging Kit, Thermo Fisher). Stained cells were analysed using a BDpathway 855 Bioimager. For Incucyte analysis, images of cells were acquired every 20 min with four images per well and IncuCyte ZOOM (Essen BioScience) software was utilized for measurement of confluency.

### Cell lysis and Western blotting

Cells were lysed and analysed as described [51, 52]. Primary antibodies were as follows: ERK2 (sc-154), SRC (sc-18), PAX3 (N19) and YAP1 (sc-101199): SCBT, Santa Cruz, CA, USA; phospho-ERK (#M8159): Sigma, St Louis, MO, USA; phospho-SRC (#2101), phospho-MLC2 (#3671), phospho-YAP (#4911): Cell Signaling, Boston, MA, USA; phospho-FAK (#44-626G and #611807), FAK (#610087): BD Biosciences, Oxford, UK; MITF (MA5-14146): Thermo Fisher, Loughborough, UK; phospho-LATS1/2 (ab111344): Abcam, Cambridge, UK. Detection was done by ECL using horse radish peroxidase-coupled secondary antibodies.

### RNA extraction, cDNA synthesis and quantitative PCR analysis

RNA was isolated using TRIZOL^®^ (Invitrogen, Carlsbad, CA, USA) and quantitative real-time PCR performed using SENSIMIX SYBR reagent (Bioline, Boston, MA, USA). Expression levels were quantified relative to glyceraldehyde 3-phosphate dehydrogenase via the Livak method. The following primer sequences were used: MITF, 5′-CCGTCTCTCACTGGATTGGT-3′, 5′-TACTTGGTGGGGTTTTCGAG-3′; TRPM1, 5′-CACCCAGAGCTACCCAACAGA-3′, 5′-CGGATATACATGGCTTTATTGG-3′; PMEL, 5′-CTGGATGGTACAGCCACCTT-3′, 5′-GGCACTTTCAATACCCTGGA-3′; TYR, 5′-CTGGAAGGATTTGCTAGTCCAC-3′, 5′-CCTGTACCTGGGACATTGTTC-3′; MLANA, 5′-TCTGGGCTGAGCATTGGG-3′, 5′-AGACAGTCACTTCCATGGTGTGTG-3′; CDK2, 5′-ATGGAGAACTTCCAAAAGGTGGA-3′, 5′-CAGGCGGATTTTCTTAAGCG-3′; CCND1, 5′-GAACTACCTGGACCGCTTCCT-3′, 5′-TTCGATCTGCTCCTGGCAGG-3′; BCL2A1, 5′-CGTAGACACTGCCAGAACACTA-3′, 5′-GGGCAATTTGCTGTCGTAGA-3′; CTGF, 5′-CGACTGGAAGACACGTTTGG-3′, 5′-ATCCCACAGGTCTTGGAACA-3′; NEGR, 5′-GGTCAGTGGATCCTCGAGTT-3′, 5′-TGGGCCATCATCTGTCACAT-3′; CYR61, 5′-TGCGAAGATGGGGAGACATT-3′, 5′-CTGTAGAAGGGAAACGCTGC-3′; *CTGF* promoter, 5′-GCCAATGAGCTGAATGGAGT-3′, 5′-CAATCCGGTGTGAGTTGATG-3′; *CYR61* promoter, 5′-AGCAAACAGCTCACTGCCTT-3′, 5′-ATGGTAGTTGGAGGGTCGTG-3′; *MITF* promoter, 5′-GCAGTTATTCGGCCATTGGA-3′, 5′-GGAAGCCCTACGAGTTTGGT-3′.

### Immunoprecipitation and ChIP

Cells were lysed [53] and immunoprecipitation was done using G-sepharose, 5 μg of goat anti-PAX3 (N19) (Santa Cruz Biotechnology) or immunoglobulin G (IgG) control (goat IgG) and analysed by sodium dodecyl sulphate-polyacrylamide gel electrophoresis and Western blotting. ChIP assays, using control IgG-specific or YAP1-specific (63.7) antibodies (both Santa Cruz Biotechnology), were performed as described previously [54]. The primer sequences used were as follows: *CTGF *promoter, 5′-GCCAATGAGCTGAATGGAGT-3′ and 5′-CAATCCGGTGTGAGTTGATG-3′; *CYR61* promoter, 5′-AGCAAACAGCTCACTGCCTT-3′ and 5′-ATGGTAGTTGGAGGGTCGTG-3′; *MITF* promoter, 5′-GCAGTTATTCGGCCATTGGA-3′ and 5′-GGAAGCCCTACGAGTTTGGT-3′.

### Luciferase assay

Cells were transfected and analysed with 0.2 μg of CMV-Renilla and with either 0.5 μg of pMAX GFP or pQCXIH-Myc-YAP5SA [25], and 1 μg of *MITF* promoter luciferase reporter constructs pGL2-333MITF or pGL2-333mut as described [26].

### Gel contraction

Plates were blocked with phosphate-buffered saline/3% bovine serum albumin (BSA) for 1 h 37 °C, washed and air dried. A total of 10^5^ fibroblasts mixed with 1.7 mg/ml collagen solution (2.3 mg/ml, Nutacon, Leimuiden, The Netherlands) were seeded, and after 1 h 100 μl DMEM/10% FCS was added After 3 days, the contracted gels were photographed and analysed using ImageJ.

### Immunofluorescence

Cells were fixed in 4% formaldehyde/0.2% Triton-X (Sigma, Dorset, UK), and after blocking (3% (w/v) in BSA), it was incubated with primary and secondary antibodies. The following antibodies were used for immunofluorescence: COLTypeI, ab88147 from Abcam; FN1, F3648 from Sigma; YAP1 sc-101199 from Santa Cruz Biotechnology; Alexa Fluor™ 594 Phalloidin, A12381, Alexa Fluor™ 488 anti-mouse, A11001 and Alexa Fluor™ 594 anti-rabbit, A-11037, from Thermo Fisher.

### Immunohistochemistry of TMA

Antigen retrieval and 3,3'-diaminobenzidine staining was performed as previously described [49]. PicroSirius Red Stain Kit was used to stain for collagen. Antibodies used in immunohistochemistry were as follows: ACTA2/α SMA, 180106 from Invitrogen; COL1, ab150681 from Abcam; FN1, F3648 from Sigma; YAP1, sc-101199 from Santa Cruz.

### Data analysis

Data from TCGA was downloaded through cBioportal. Additional data were obtained from GSE22153 [27] and analysed by GEO2R. mRNA expression values were *z*-score transformed and hierarchical clustering was performed using Heatmapper [55]. The Average Linkage method was used for clustering and distance measurement was calculated using the Pearson's method. For survival analyses TCGA patient data was extracted using OncoLnc and analysed in OncoLnc or GraphPad Prism version 6 (San Diego, CA, USA). All experiments are from a minimum of three biological replicates. Shown are *n* = 3 experiments, analysed using non-normalized one-way analysis of variance (ANOVA) with Tukey’s post hoc test in GraphPad Prism version 6. *P* values of <0.05 were considered statistically significant and all *p* values are stated in the figure legends together with the standard error of the mean (SEM).

## Electronic supplementary material

Supplementary Figures and Table(PDF 3456 kb)
