# Supplementary material for: Collagen abundance controls melanoma phenotypes through lineage-specific microenvironment sensing
Source: Oncogene. 2018 Mar 16;37(23):3166–82. doi: 10.1038/s41388-018-0209-0 (PMC5992128; doi:10.1038/s41388-018-0209-0)
Supplement: Supplementary file 1 — Supplementary Figures and Table(PDF 3456 kb) [file 41388_2018_209_MOESM1_ESM.pdf]

Supplementary Information (SI)

Collagen abundance controls melanoma phenotypes through lineage specific microenvironment sensing

Miskolczi et al.

Supplementary Data

Figure S1, related to Figure 1
YAP/TAZ localization depends on matrix stiffness

Figure S2, related to Figure 1
SRC, FAK and ROCK regulate YAP/TAZ localization

Figure S3, related to Figure 2
Collagen deposition correlates with fibronectin expression

Figure S4, related to Figure 3
YAP/TAZ and PAX3 co-localise in melanoma cells

Figure S5, related to Figure 4
YAP regulates melanoma cell proliferation

Figure S6, related to Figure 7
Hierarchical clustering of TCGA melanoma dataset

Figure S7, related to Figure 7
Differentiation and proliferation gene expression correlate with poor survival in melanoma patients

Table S1, related to Figure 2
Patient Characteristics of TMA

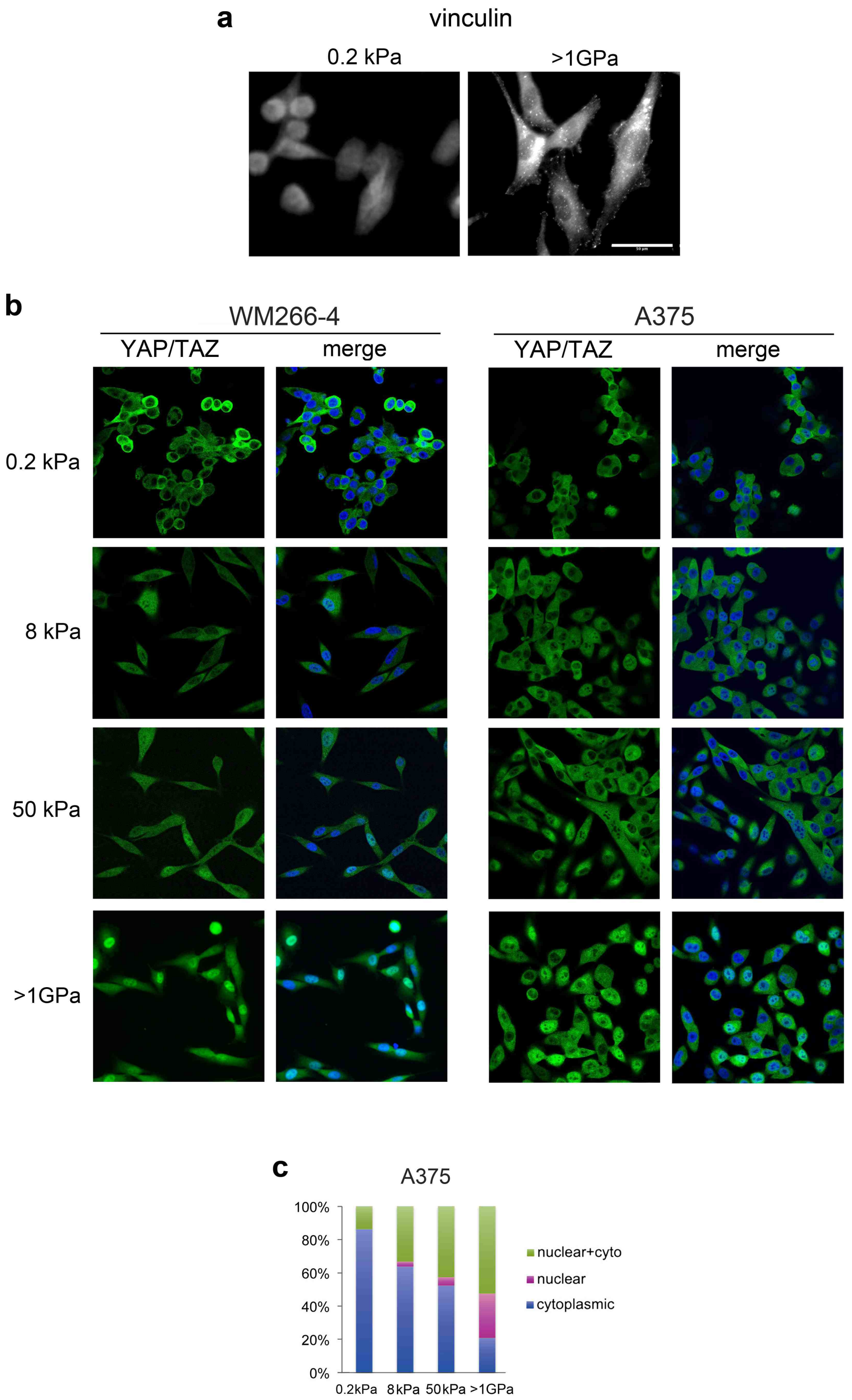

Figure S1, related to Figure 1. YAP/TAZ localization depends on matrix stiffness

(a) Immunofluorescence analysis of vinculin in WM266-4 cells cultured on collagen with the indicated stiffness degrees for 72h.

(b) Immunofluorescence analysis of YAP/TAZ localisation in WM266-4 and A375 cells cultured on collagen with the indicated stiffness degrees for 72h.

(c) Quantification of YAP/TAZ localisation in A375 cells cultured on collagen with the indicated stiffness degrees for 72h (n = 3 experiments; n = 100 cells)

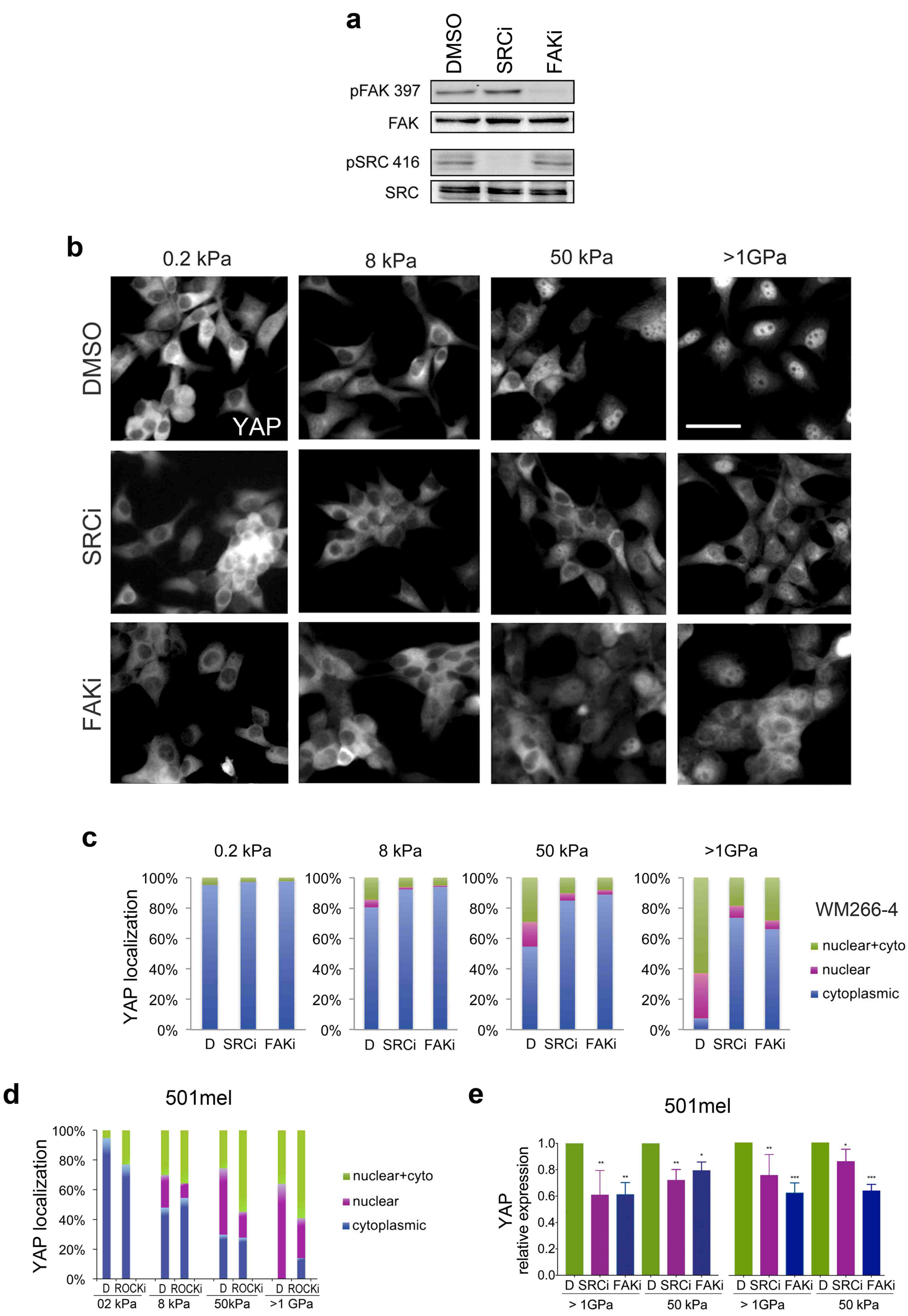

Figure S2, related to Figure 1. SRC, FAK and ROCK regulate YAP/TAZ localisation

(a) Western blot analysis of pFAK, and pSRC in 501mel cells treated with 1μM AZD0530 (SRCi), 1μM PF562271 (FAKi) or DMSO for 24h.
(b) Immunofluorescence analysis of YAP/TAZ localisation in 501mel cells cultured on collagen with the indicated stiffness degrees and treated with 1μM (SRCi, 1μM FAKi or DMSO for 24h. Quantification, see Figure 1f.
(c) Quantification of YAP1/TAZ localisation in WM266-4 cells treated with 1μM SRCi, 1μM FAKi or DMSO for 24h (n = 3 experiments; n = 100 cells).
(d) Quantification of YAP1/TAZ localisation in 501mel cells treated with 10μM Y-27632 or DMSO for 48h (n = 3 experiments; n = 100 cells)
(e) qRT-PCR for YAP in 501mel cells treated with 1μM SRCi, 1μM FAKi or DMSO for 48h.

a

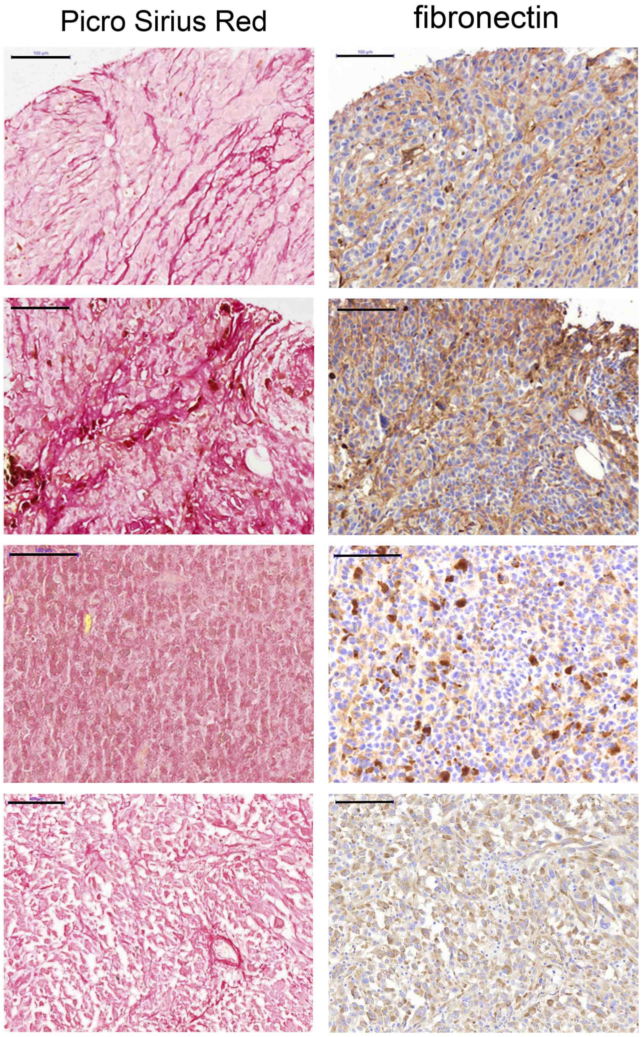

b

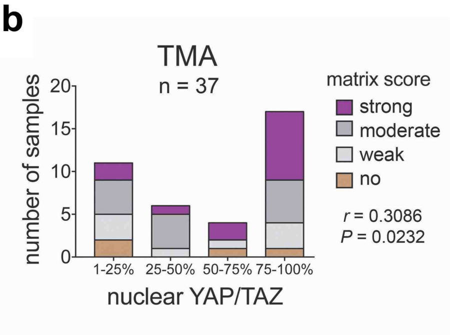

Figure S3, related to Figure 2. Collagen deposition correlates with fibronectin expression

(a) Immunohistochemistry of tissue microarray of primary and metastatic melanocytic lesions stained with PicroSirius Red (for collagen) and fibronectin (FN1) antibody. Scale bars represent 100μm.

(b) Correlation analysis of YAP/TAZ nuclear localisation as indicated in (%) and matrix abundance characterised by a matrix score. p=0.0232 and r=0.3086.

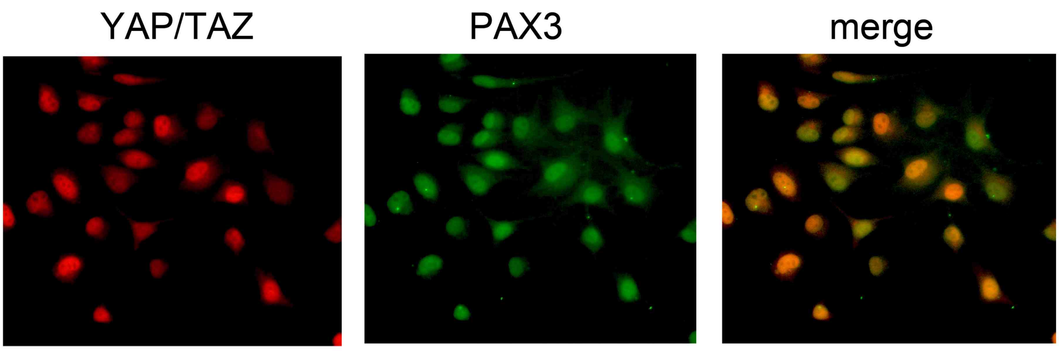

Figure S4, related to Figure 3. YAP/TAZ and PAX3 co-localise in melanoma cells

Immunofluorescence analysis of YAP/TAZ and PAX3 in 501mel cells.

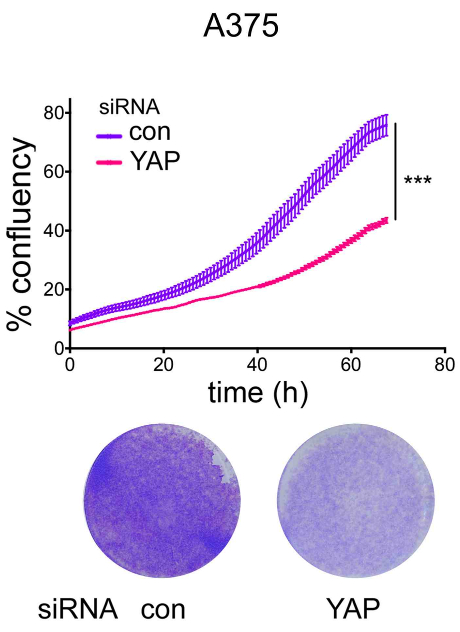

Figure S5, related to Figure 4. YAP regulates melanoma cell proliferation

Incucyte analysis and crystal violet staining to measure cell growth (confluency) of A375 cells upon YAP SMART*pool* siRNA treatment

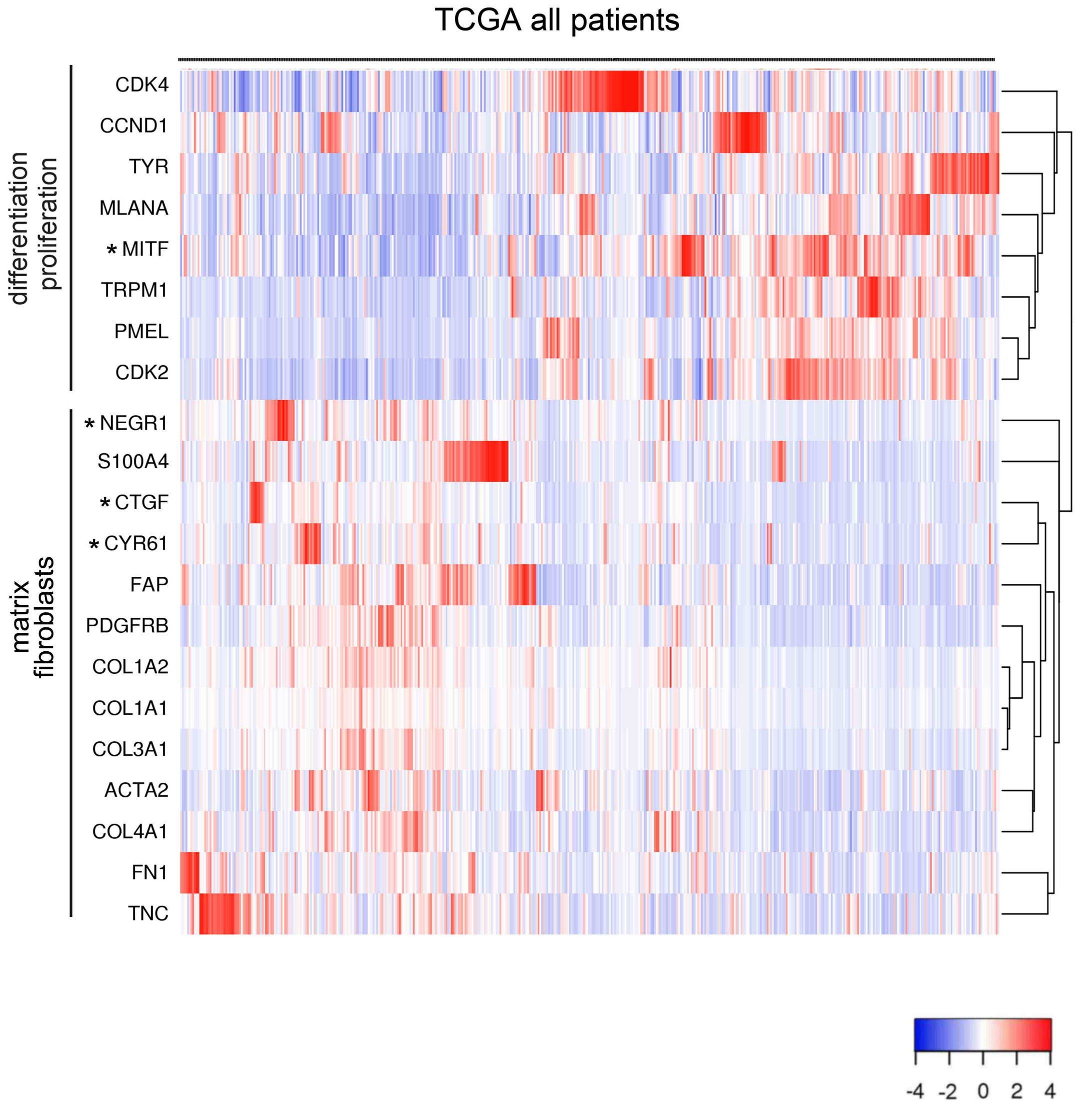

Figure S6, related to Figure 7. Hierarchical clustering of TCGA melanoma dataset

Hierarchical clustering of gene expression data derived from TCGA melanoma patients. The two main clusters represent fibroblast/TGFβ and differentiation/proliferation signatures

a

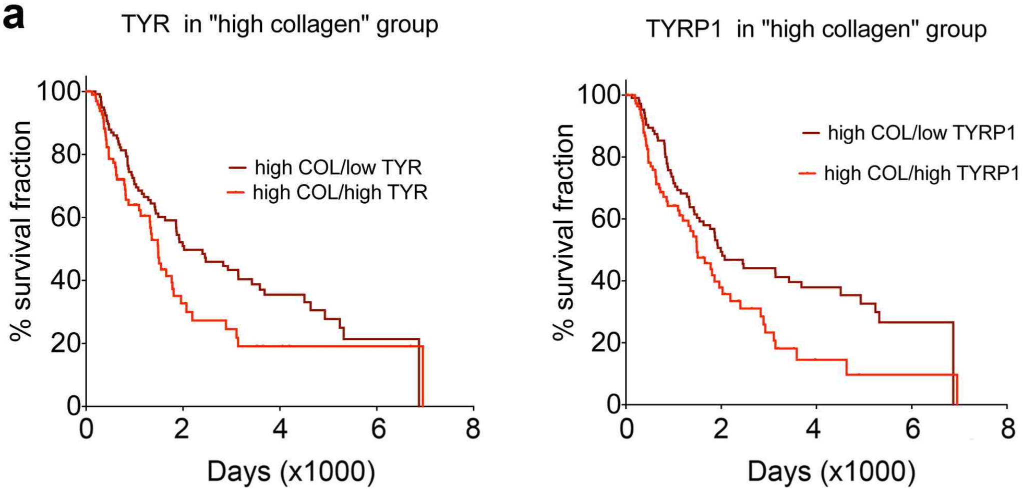

b

COL1A1	TRPM1	Median survival Estimate	Ratio	95% Confidence Interval Lower Bound	Upper Bound
HIGH	LOW	66.710	0.704	0.4918	1.010
	HIGH	47.006	1.419	0.9905	2.033

COL1A1	TYR	Median survival Estimate	Ratio	95% Confidence Interval Lower Bound	Upper Bound
HIGH	LOW	66.513	0.7349	0.5122	1.055
	HIGH	48.881	1.3610	0.9483	1.952

COL1A1	TYRP1	Median survival Estimate	Ratio	95% Confidence Interval Lower Bound	Upper Bound
HIGH	LOW	65.526	0.746	0.5226	1.066
	HIGH	48.854	1.340	0.9379	1.913

COL1A1	CDK4	Median survival Estimate	Ratio	95% Confidence Interval Lower Bound	Upper Bound
HIGH	LOW	80.723	0.5383	0.3761	0.7704
	HIGH	43.453	1.858	1.298	2.659

Figure S7, related to Figure 7. Differentiation and proliferation gene expression correlate with poor survival in melanoma patients

(a) Kaplan-Meier analyses using the TCGA melanoma dataset. Differences in overall survival for high and low expression of TYR or TYRP1 respectively in the 'high collagen' group (n = 228) are shown. For TYR: Hazard ratio (HR) log-rank 1.453 for high TYR (CI 95% 0.9973 to 2.117); p (log-rank) = 0.0347; for TYRP1: Hazard ratio (HR) log-rank 1.579 for high TYRP1 (CI 95% 1.093 to 2.282); p (log-rank) = 0.0086.

(b) Median survival in months in the 'high collagen' group (n = 228) using the TCGA melanoma dataset is shown.

Patient number	Sex	Age	Organ	TNM	Stage
1	M	71	Skin	II	T4N0M0
3	F	41	Skin	II	T4N0M0
4	F	62	Skin	II	T4N0M0
5	F	61	Skin	II	T4N0M0
6	M	60	Skin	IIC	T4BN0M0
7	M	25	Skin	I	T2N0M0
10	M	80	Skin	II	T4N0M0
11	M	51	Skin	III	T4N1M0
12	M	69	Skin	IIA	T2N0M0
13	M	41	Skin	II	T3N0M0
14	M	37	Skin	II	T4N0M0
15	M	61	Skin	III	T4N2M0
18	M	52	Skin	III	T4N1M0
20	F	54	Skin	II	T4N0M0
21	M	42	Skin	IV	T3N2M1
22	F	42	Skin	IIB	T4N0M0
23	M	49	Skin	IIA	T2BN0M0
24	M	71	Skin	II	T4N0M0
25	F	83	Skin	I	T2N0M0
26	M	51	Skin	IIB	T4N0M0
30	M	66	Skin	II	T4N0M0
34	F	72	Skin	II	T4N0M0
37	M	49	Skin	III	T4N2M0
39	F	72	Vulva	III	T4N0M0
40	F	57	Vulva	III	T4N0M0
41	F	38	Vulva	III	T4N0M0
42	F	44	Vulva	III	T4N0M0
45	F	38	Rectum	–	–
48	F	52	Rectum	II	T4N0M0
49	F	84	Rectum	II	T4N0M0
50	F	67	Rectum	II	T4N0M0
54	F	54	Rectum	III	T3N2M0
56	M	55	Stomach	–	–
57	M	55	Stomach	–	–
59	M	64	Esophagus	–	–
60	M	71	Intestine	–	–
61	M	73	Intestine	–	–
62	F	70	Oral cavity	II	T4N0M0
63	F	63	Lymph node	–	–
66	M	44	Lymph node	–	–
70	F	40	Lymph node	–	–
71	M	70	Lymph node	–	–
72	M	56	Lymph node	–	–
73	M	68	Lymph node	–	–
76	F	61	Lymph node	–	–
78	F	43	Lymph node	–	–
79	M	73	Lymph node	–	–
80	F	56	Lymph node	–	–
81	F	49	Lymph node	–	–
82	F	41	Lymph node	–	–
86	F	10	Skin	–	–
91	M	20	Skin	–	–
92	F	2	Skin	–	–
94	M	46	Skin	–	–

Table S1, related to Figure 2. Patient Characteristics of TMA

The age, sex, organ site and stage including TNM of the samples included in the TMA analysis are shown.
